# Rice Phyllosphere *Bacillus* Species and Their Secreted Metabolites Suppress *Aspergillus flavus* Growth and Aflatoxin Production *In Vitro* and in Maize Seeds

**DOI:** 10.3390/toxins10040159

**Published:** 2018-04-16

**Authors:** Subbaiah Chalivendra, Catherine DeRobertis, Jorge Reyes Pineda, Jong Hyun Ham, Kenneth Damann

**Affiliations:** Department of Plant Pathology and Crop Physiology, Louisiana State University AgCenter, Baton Rouge, LA 70803, USA; cderobertis@agcenter.lsu.edu (C.D.); jar45788@uga.edu (J.R.P.); jham@agcenter.lsu.edu (J.H.H.); kdamann@agcenter.lsu.edu (K.D.)

**Keywords:** aflatoxin, interspecific biocontrol, rice-associated *bacilli* (RABs), seed aflatoxin contamination, field trials, tip lysis, lipopeptides, fungistasis, anti-aflatoxigenic activity, hemolytic activity

## Abstract

The emergence of super-toxigenic strains by recombination is a risk from an intensive use of intraspecific aflatoxin (AF) biocontrol agents (BCAs). Periodical alternation with interspecific-BCAs will be safer since they preclude recombination. We are developing an AF-biocontrol system using rice-associated *Bacilli* reported previously (RABs). More than 50% of RABs inhibited the growth of multiple *A. flavus* strains, with RAB4R being the most inhibitory and RAB1 among the least. The fungistatic activity of RAB4R is associated with the lysis of *A. flavus* hyphal tips. In field trails with the top five fungistatic RABs, RAB4R consistently inhibited AF contamination of maize by Tox4, a highly toxigenic *A. flavus* strain from Louisiana corn fields. RAB1 did not suppress *A. flavus* growth, but strongly inhibited AF production. Total and HPLC-fractionated lipopeptides (LPs) isolated from culture filtrates of RAB1 and RAB4R also inhibited AF accumulation. LPs were stable *in vitro* with little loss of activity even after autoclaving, indicating their potential field efficacy as a tank-mix application. *A. flavus* colonization and AF were suppressed in RAB1- or RAB4R-coated maize seeds. Since RAB4R provided both fungistatic and strong anti-mycotoxigenic activities in the laboratory and field, it can be a potent alternative to atoxigenic *A. flavus* strains. On the other hand, RAB1 may serve as an environmentally safe helper BCA with atoxigenic *A. flavus* strains, due its lack of strong fungistatic and hemolytic activities.

## 1. Introduction

Aflatoxin (AF), a mycotoxin made by *Aspergillus flavus*, is the most dangerous crop contaminant due to its acute carcinogenicity. AF is strictly regulated in the US (20 ppb according to FDA, the Food and Drug and Administration) and affects the marketability of maize and other commodities from time to time. It poses a more serious threat to food safety and security in developing countries. Breeding efforts have identified only partial resistance in maize to the ear rot caused by *A. flavus* [1]. Discovering the ability of non-aflatoxigenic *A. flavus* strains to reduce AF contamination in field-grown maize [2] opened up a potent strategy for AF mitigation. Currently, biocontrol by atoxigenic *A. flavus* strains is the only effective control measure. However, there are two key concerns in the use of intraspecific biocontrol strains: (1) the efficacy of biocontrol strains is seasonal, and effective against only a limited range of toxigenic strains whose populations keep changing periodically; and (2) the inhibition of AF biosynthesis requires a physical contact of the biocontrol strain with the toxigenic one within the first 24 h of spore germination of the latter (“touch inhibition” [3]). This demands a routine deployment of atoxigenic strains to maintain inoculum and assured biocontrol. The steady contact may enhance the risk of developing super-toxigenic strains by sexual recombination between toxigenic lines and the typically more vigorous biocontrol strains [4,5,6]. An inter-specific biocontrol agent (BCA) precludes mating and, thereby, the emergence of hyper-virulent toxigenic strains. If the BCA needs no direct contact with the toxigenic strains for its activity, the risk of even horizontal gene transfer is minimized. Furthermore, many bacterial BCAs show antifungal activities against a wide range of pathogens.

Even before the discovery of atoxigenic *A. flavus* strains, diverse bacteria and yeasts colonizing the bulk soil, rhizosphere, and phyllosphere were reported to show strong AF biocontrol activity in vitro (Reviewed in [7,8,9]). However, only phyllosphere colonizers are shown to reduce AF in field trials, since these organisms occupy and compete for the same niche as *A. flavus* [7]. The phyllosphere presents a harsher environment than the soil due to rapidly-fluctuating temperatures, low humidity, nutrient scarcity, and UV irradiation, and these conditions support a limited niche complexity [10]. On the other hand, interspecific competition among phyllosphere microorganisms is stronger than in soil microbes. Thereby, the phyllosphere favors the evolution of antagonistic activities against competing microbes. For example, among 892 bacterial isolates from soil and aerial parts of cotton, only six from cotton balls reduced AF in a laboratory bioassay, and only four of these in field trials [11]. Therefore, our search for novel AF biocontrol agents, interspecific in particular, is focused on phyllosphere microbes. Here, we present our work on commensalistic *Bacillus* and related strains isolated from rice leaves (RABs; [12]). These bacteria were shown to be fungi-, as well as bacteriostatic, and ameliorate both fungal and bacterial blight diseases in rice, previously [12]**.** As genetic and environmental differences may not be conducive for the BCA strains from one host to establish on a heterologous host species, live BCAs need to be tested for their ability to colonize in densities that are necessary to control aerial pathogens under field conditions [13]. Many *Bacillus* spp. secrete a wide array of bioactive metabolites, such as polyketides, peptides, and siderophores, thus offering an ex vivo method of pathogen control [14,15]. We tested the field control of AF in maize using live RABs. Further, we investigated the anti-aflatoxigenic activity and environmental stability of isolated lipopeptides, properties important for their potential use in the field.

## 2. Results

### 2.1. Many Rabs Showed Significant Fungistatic Activity on A. flavus Strains

**Figure 1:**
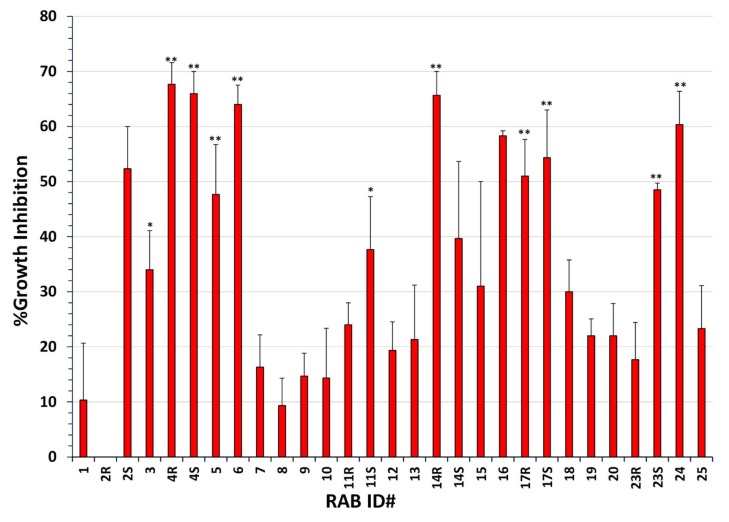
Fungistatic effects of RAB isolates on *A. flavus*. The percentage growth inhibitory zone for each of the four *A. flavus* strains was calculated as described in Section 5.2. Three plates were used per every RAB strain and the experiment was repeated twice for each *A. flavus* strain. The values shown are average + standard error of growth inhibition in all four *A. flavus* isolates. ** = RABs with highly significant (*p* ≤ 0.01) growth inhibition; * = RABs with significant (*p* ≤ 0.05) growth inhibition.

The magnitude of radial growth inhibition in four different toxigenic *A. flavus* strains (NRRL3357, 53, *A. flavus nor* mutant, and Tox4) by all 29 RABs was measured by inoculating the fungus at the center and the RABs in three or four corners of the plate. Although the extent of inhibition by a few RABs depended on the *A. flavus* strain used and the composition of the medium, more than 68% of RABs showed ≥20% antagonism to *A. flavus* and nearly a third showed >50% growth inhibition (Figure 1). Twelve RABs showed statistically significant inhibition, of which ten were highly significant in their effect (Figure 1). The antagonistic effects of individual RABs on *A. flavus* growth were different from their effects on *Rhizoctonia solani* reported earlier [12]. For example, all three *Lysinibacillus* strains (RAB1, RAB5, and RAB12) showed no measurable growth inhibition of *R. solani* [12], while RAB5 was effective (~50% growth inhibition) on *A. flavus* (Figure 1). RAB6 and RAB17S were among the few isolates that showed strong growth inhibition in both fungi.

### 2.2. RABs Differ in Fungistasis and Anti-Mycotoxigenic Activitivities

**Figure 2:**
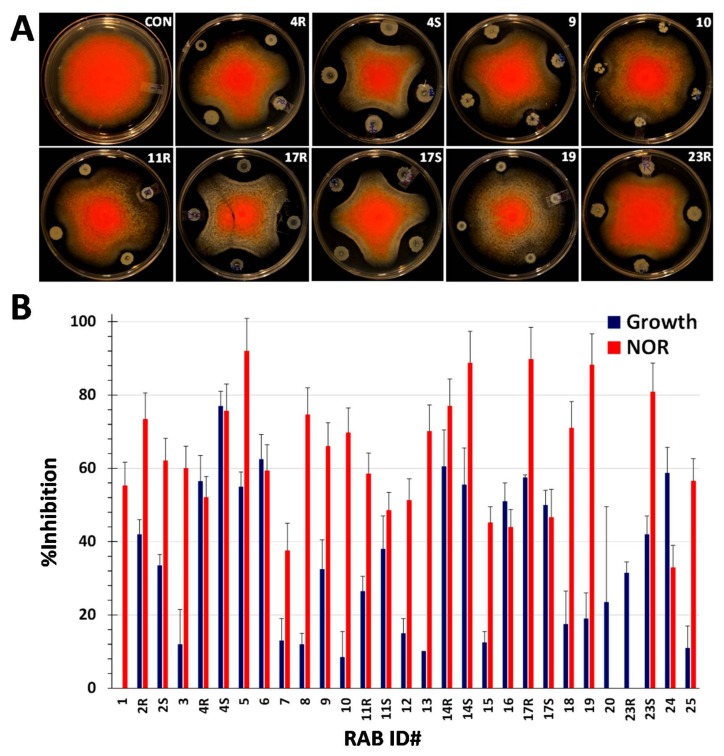
Inhibition of colony growth and NOR production by RABs. (**A**) The *nor* mutant and RABs were spot-inoculated in three replicate plates as described in Section 5.2 and Section 5.4. Plates were photographed after a 4 d incubation at 30 °C in the dark. Only nine RABs are shown to illustrate the diversity of inhibition patterns. RABs such as 17R inhibited both growth and NOR equally and a few others showed neither activity (e.g., 23R). Some RABs inhibited preferentially either the growth (e.g., 17S) or NOR (e.g., 19). (**B**) Inhibition of colony growth and NOR accumulation are shown for all 29 RAB-*nor* interactions. Except for RAB7, RAB20, and RAB23R, the rest of the RABs showed significant reduction in NOR, of which 18 were highly significant as determined by Tukey’s HSD. Against this, fungistasis was significant only in 15 RABs. These two activities were poorly correlated, indicating that they may be independently controlled.

One of the strains used in growth inhibition assays is *A. flavus nor* mutant. The mutant is blocked in AF biosynthesis after the formation of the first stable and committed intermediate, norsolorinic acid (NOR; [16]). *nor* mutants in *A. flavus* and *A. parasiticus* have been used as visual and quantitative reporters of AF both in growth media and infected seeds [17,18] and to analyze AF-biocontrol activities of yeasts [19]. The mutant allowed us to measure both fungistatic and anti-aflatoxigenic activities of all 29 RABs within the same experiment. As shown in Figure 2, RABs showed varied effects on the radial growth and NOR production by the fungus. Many isolates that showed considerable inhibition of NOR were poorly fungistatic. Conversely, a few RABs that strongly impacted growth were less effective in reducing NOR content. A majority of RABs showed strong suppression of NOR but low, or no, fungistasis, accounting for a lack of correlation between the two activities.

### 2.3. Field Testing of Top Five Rabs on AF Control in Maize

**Figure 3:**
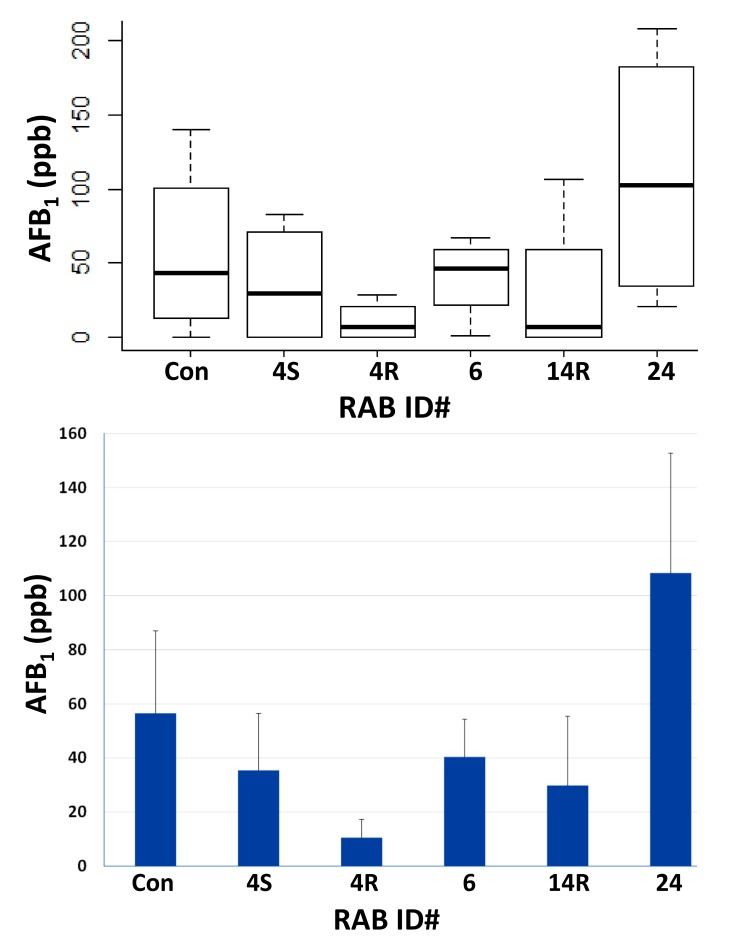
Field efficacy of RABs on aflatoxin mitigation in *A. flavus-*infected maize. Silks were pre-inoculated with indicated RABs and, after 3 d, the same silks were sprayed with Tox4 conidia as described in Section 5.3. Four replicate plots were used in the analysis. The distribution of seed AF values from the water control (**Con**) and RAB treatments is presented as a box plot in the top panel. The bottom panel shows the average +SE measures of the data. The differences are not statistically significant as determined by Tukey’s HSD, since infection was poor (resulting in 0 ppb of AF) in one of the control plots.

The field efficacy of the five RABs, viz., 4R, 4S, 6, 14R, and 24, that showed 60% or greater inhibition of *A. flavus* growth in vitro (Figure 1) was tested on AF-biocontrol in maize seeds. RAB4S and RAB6 reduced seed AF to some extent, but RAB4R was more efficacious in AF biocontrol (Figure 3). In addition to Tox4, *A. flavus* strain 53 (also used in fungistasis studies) was tested in the same field trial. However, the negative control showed poor infectivity, which is not uncommon in AF-field trials (more below). Nevertheless, AF levels in RAB4R-treated ears were consistently the lowest of all five RABs (data not shown). The trial was repeated in the following season to test the effects of only RAB4R on AF contamination by 53. The strain showed high infectivity only in one of the four replicate plots. Still, the average seed AF in the control was 15-fold greater than in RAB4R pretreated ears (Figure S1). In our future field trials, we plan to include RABs that showed strong inhibition of both growth as well as NOR (e.g., 14R and 17R; Figure 2) along with RAB4R, which was selected solely based on its superior fungistatic activity. Furthermore, those RABs that strongly inhibited AF synthesis without affecting *A. flavus* growth (Figure 2) may work as helper BCAs with atoxigenic *A. flavus* strains or with other RABs showing strong fungistatic activity, but poor aflatoxigenic activity, and provide improved AF biocontrol of multiple toxigenic strains. We will identify the best cocktail for field trials after testing their compatibilities in the laboratory.

In addition to consistent and strong reductions of seed AF levels by RAB4R in field trials, the bacterium proved to be better than intraspecific biocontrol strains in suppressing AF levels in vitro. As a part of another study, we compared NOR and AF biocontrol by major atoxigenic *A. flavus* strains viz., 19, AF36, NRRL 21882 (Afla-Guard), 51, and 49 with that of RAB4R in YES (yeast extract-sucrose) medium using *nor* and VCG8 (vegetative compatibility group 8; a toxigenic strain isolated from local corn fields, [20]). Most intraspecific biocontrol strains gave ≥90% reduction of AF or NOR in these assays but the actual AF content was 10–20 fold greater than FDA-regulated levels. In contrast, RAB4R gave near complete elimination (~10–20 ppb; Figure S2; data not shown). Therefore, we have focused our analysis on RAB4R, a *Bacillus methylotrophicus* strain in comparison to RAB1, a strain belonging to *Lysinibacillus sphaericus* (formerly known as *Bacillus sphaericus* [21]). RAB1 is poorly fungistatic to both *A. flavus* (Figure 1) and *R. solani* [12] in commonly-used solid media (glucose minimal medium, GMM; potato-dextrose agar, PDA; or corn meal agar, CMA) and inhibited *A. flavus* growth only in the blood agar base (BAB), a protein-rich medium. 

### 2.4. RAB1 Shows Anti-Aflatoxigenic Activity in Spite of Lacking Fungistatic Activity

**Figure 4:**
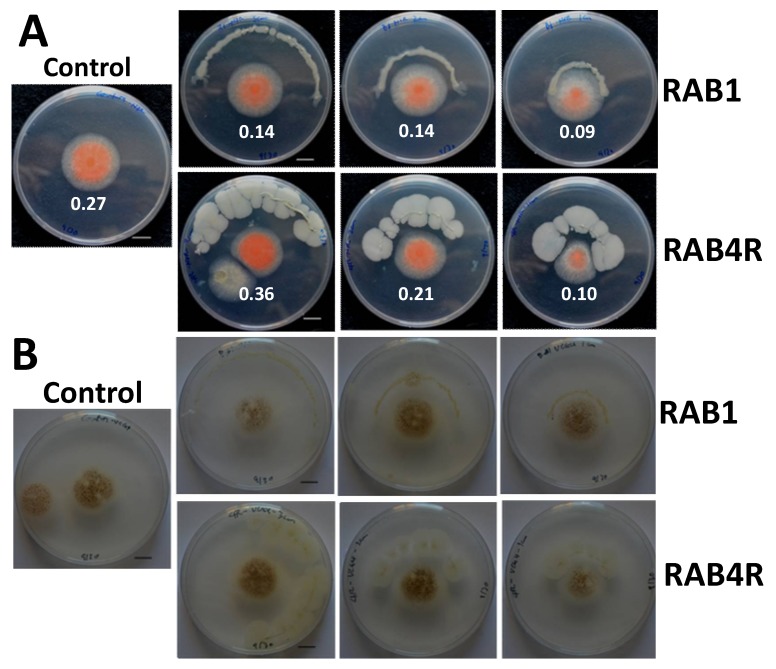
Proximity, but not touch, is required for the biocontrol action of RABs. The *nor* mutant (panel **A**) or the aflatoxigenic isolate Tox 4 (panel **B**) was spot-inoculated at the center of corn meal agar plates and grown for 3 d at 30 C. RAB 1 and RAB4R were inoculated at 3, 2, and 1 cm away from the center in an arch shape. RAB1 was slow-growing and showed no fungistatic activity. However, it suppressed NOR accumulation better than the fungistatic RAB4R. NOR was extracted from a 12 mm plug from the center of the colony and measured spectrophotometrically. A_560_ (absorbance at 560 nm) values are shown in white below the *nor* colony in each plate. Tox 4 plates were stained for AF using ammonia vapors as described in [23] (panel **B**).

A zone of *A. flavus* growth inhibition around many RABs implies that a physical contact with the fungus is not essential for the biocontrol effect. It is also strongly indicative of the involvement of diffusible antifungal metabolites in the biocontrol reaction. We tested this possibility, by growing RAB4R and RAB1 at different distances from *A. flavus*. The *nor* mutant and the toxigenic isolate, Tox4, were tested in these experiments. RAB1 and RAB4R were plated at 3, 2, and 1 cm away from the center in an arch shape (Figure 4). RAB4R grew rapidly on CMA, while RAB1 growth was slow, as was observed on other common fungal media. Inhibition of growth and NOR or AF production was inversely related to the distance between *A. flavus* and RAB4R, indicating that it secretes diffusible biocontrol-active metabolites into the medium. RAB1, in spite of its poor growth or lacking fungistatic activity, showed a clear suppression of NOR and AF production, even from the farthest distance tested (3 cm). This indicated that the presumptive antifungal metabolites secreted by RAB1 are predominantly anti-mycotoxigenic in effect and may diffuse faster than those made by RAB4R. An isolate of *L. sphaericus* produces volatile antimicrobial metabolites that are inhibitory to phytopathogenic bacteria and fungi [22].

### 2.5. RAB4R Effects on Hyphal Growth

**Figure 5:**
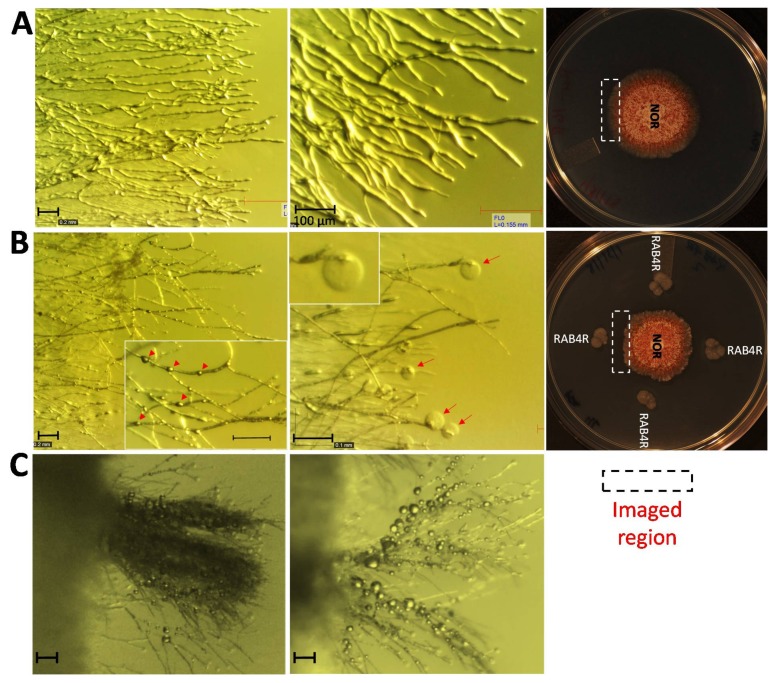
RAB4R induces intercalary cell swelling, tip lysis, abnormal branching, and increased surface hydrophobicity in *A. flavus*. (**A**) The edge of *A. flavus* colony at 30× (left panel) and 63× (right panel) magnification showing uniformly-growing well-rounded hyphal tips. (**B**) The edge of *A. flavus* colony from the growth inhibition zone induced by RAB4R at 30× magnification, showing an irregular growing edge with hyphae showing cell swelling (left panel). The inset show 63× magnification to highlight swollen cells marked by arrow heads. The right panel shows hyphal tip lysis marked by red arrows. The inset show 63× magnification to highlight a lysed hyphal tip. (**C**) Abnormal hyphal branching and increased hydrophobicity are indicated by accumulation of water droplets on the hyphal surface in the inhibition zone. The scale bar in each panel is equal to 100 μm.

We looked at the mode of action of RAB4R fungistasis by imaging the hyphal morphology in the inhibitory zone. As *A. flavus* hyphae approach RAB4R (5.2 ± 0.2 mm), their tips lyse leading to the cessation of growth (Figure 5). In addition to tip lysis, swelling of intercalary cells, hyper-branching and increased hydrophobicity of hyphae were also observed in the inhibitory zone (Figure 4). These modifications appear to be common to filamentous fungi in response to growth-inhibitory antifungal metabolites secreted by bacterial and fungal BCAs [24,25]. Unlike RAB4R, RAB1 failed to induce any fungicidal effects even in the BAB medium, where it showed fungistatic effects (data not shown).

### 2.6. Total and HPLC-Fractionated Lipopeptides Provide Effective AF Biocontrol

**Figure 6:**
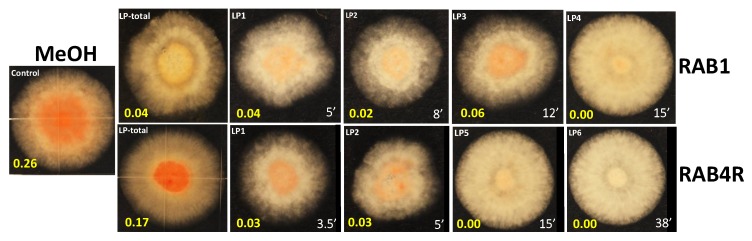
RABs secrete biocontrol-active lipopeptides (LPs) into the growth medium. RAB1 and RAB4R were grown in LB to lag phase. LPs were extracted from cell-free culture medium by acid precipitation, solubilized in methanol, concentrated and separated by HPLC as described in Section 5.6. A 50 μL aliquot of each of total extract (LP-total) or concentrated HPLC fraction was infused into PDA plates and inoculated with the *nor* mutant. Methanol (MeOH) was used as a negative control. The fractions are serially numbered by the time of their elution shown in white at the bottom right of each image. NOR values (averages of three replicates) estimated as absorbance at 560 nm are shown in yellow at the bottom left in each image. The variability among replicates was below 10%. Only fractions that gave a significant reduction of NOR are shown here. LP7 of RAB4R eluted at 49 min also gave full suppression of NOR similar to LP6 (data not shown).

Both *B. methylotrophicus* and *L. sphaericus* are known to exert biocontrol effects by the production and secretion of antifungal metabolites, including lipopeptides [21,26,27,28]. Lipopeptides (LPs) were extracted from culture filtrates of RAB1 and RAB4R and fractionated by HPLC (Figure S3). We tested crude LPs and HPLC fractions for their NOR-suppressive activity by infusing the extracts into the medium (Figure 6). LPs from both isolates reduced the accumulation of NOR. The greater reduction of NOR by individual HPLC fractions (e.g., LP5 and LP6 in RAB4R) than total LP extracts (LP_total_; Figure 6) may be due to their enhanced concentration or, alternatively, the presence of AF synthesis stimulants in RAB4R extracts.

### 2.7. Temperature and pH Stability of Lipopeptide Extracts

**Figure 7:**
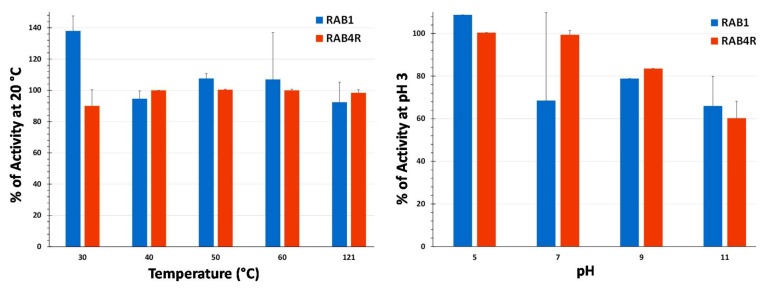
Temperature and pH stability of total LP extracts from RAB1 and RAB4R. The stability of LP preparations was tested as described in Section 5.7. The values are averages +SE of two replicated experiments.

The stability of total LPs was tested by exposing them to high temperatures, including high pressure, during autoclaving and assaying for the repression of NOR synthesis as described in Materials and Methods. Both RAB1 and RAB4R preparations showed stability with minimal loss of activity even after autoclaving (Figure 7). pH stability, also assayed using the *nor* mutant, indicated that LPs from both RAB1 and RAB4R maintain 60–70% of activity even at high alkalinity (Figure 7). Cell-free preparations of lipopeptides from many *Bacillus* spp. are remarkably stable even after prolonged (10 d) exposure to high temperatures, salinity, and extreme pH (e.g., [29]).

### 2.8. Postharvest AF Contamination in RAB1 and RAB4R Pretreated Maize Seeds

**Figure 8:**
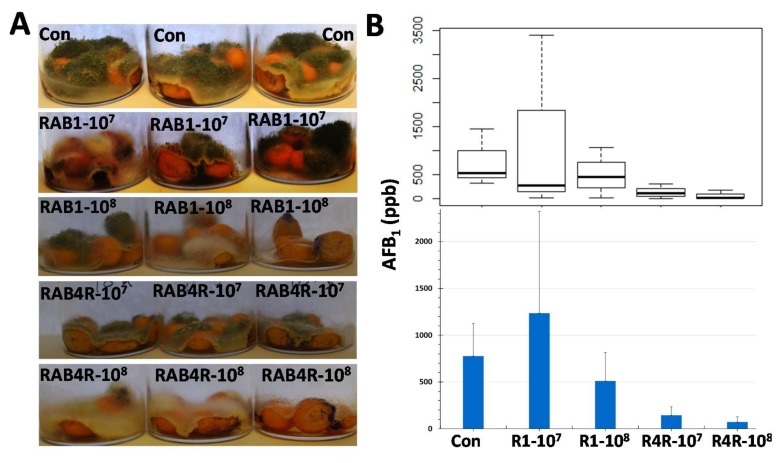
Mitigation of post-harvest *A. flavus* infection and AF contamination of maize seeds by RAB1 and RAB4R. Surface-sterilized seeds of the inbred Va35 were incubated with 10^7^ or 10^8^ cells/mL of RAB1 and RAB4R or 5% glycerol (the carrier) for 24 h. Bacteria were removed and incubated with 10^6^/mL conidia of NRRL3357 in glycerol for 7 d. (**A**) *A. flavus* growth on glycerol (Control)-, RAB1-, or RAB4R-preincubated seeds. (**B**) Seed AF was extracted on the seventh day and estimated by HPLC. The box plot in the top panel shows the spread of the data and the bar graph in the bottom panel shows the averages +SE.

*A. flavus* colonizes and contaminates both developing and stored (mature dry) maize seeds with AF. We tested the efficacy of RAB1 and RAB4R in reducing postharvest AF contamination of maize seeds, by pretreating Va35 seeds with the two RABs before inoculating them with *A. flavus* as described in Material and Methods. Although both RABs showed AF biocontrol, RAB4R was more effective and consistent in containing the mold and AF production at both 10^7^ cells/mL and 10^8^ cells/mL concentrations (Figure 8). RAB1 and RAB4R were also effective in reducing AF levels in a commercial hybrid (Pioneer 2089YHR, DuPont, Wilmington, DE, USA). However, the fungus made much lower AF (>1/10th) in the hybrid than that observed in the inbred Va35 (data not shown). 

### 2.9. Hemolytic Activity of Rabs

**Figure 9:**
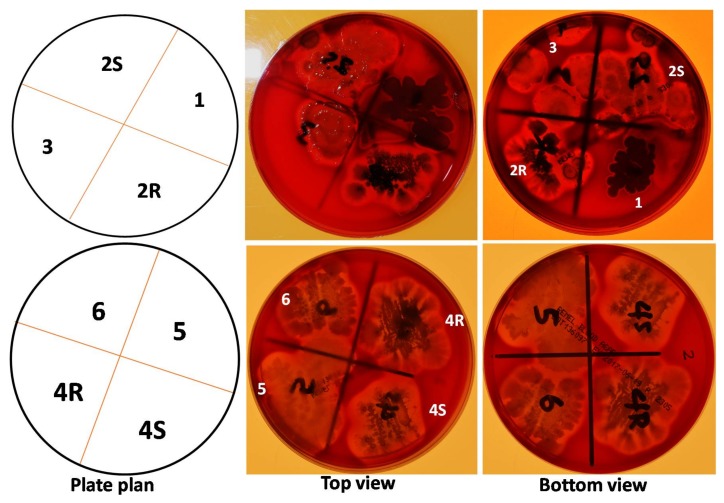
RAB1 lacks hemolytic activity. Hemolytic activity of RABs was tested by growing 4 RABs/plate for 2 d. A clearing (halo) around the colony is a due to the lysis of red blood cells by surfactin-like compounds secreted by the bacteria. Hemolytic activity was detected in all RABs, except in RAB1, in both replicate plates tested. Only the assay with first eight RABs is shown in the figure.

The hemolytic activity of all 29 isolates was analyzed using blood agar plates. RAB1 uniquely showed no lysis of erythrocytes, while all other RABs, including RAB4R showed hemolytic activity in both replicate plates (Figure 9; Figure S4). This feature makes RAB1, or its LPs, safe to non-target organisms, including humans. 

## 3. Discussion

### 3.1. Bacillus Strains from the Phyllosphere of a Non-Host Crop Strongly Inhibit A. flavus Growth and AF Synthesis Both In Vitro and in the Host

One of the main concerns in developing a biocontrol system for a plant pathogen is the uncertain ability of a putative BCA to colonize target plant hosts, particularly if the BCA is sourced from a non-host or, even worse, if it is from a different niche, such as the soil or a non-plant organism [7,10,13,30]. Since *A. flavus* does not colonize rice (even though grown in the same Louisiana soils as that of maize), we verified and found that >30% of RABs induced strong (50% or greater) inhibition of colony growth in four *A. flavus* strains (Figure 1). In vitro screening allows a rapid testing of many strains. However, the in vitro effects may not always translate to *in planta* efficacy [31]. Hence, our subsequent focus was to (1) test promising isolates and identify the most field-efficacious RAB(s), and (2) characterize further (e.g., mode of action) those isolates showing proven efficacy in the field. Of the top five isolates tested, RAB4R was consistently effective in restricting AF contamination in field trials (Figure 3 and Figure S1). Other RABs were equal to RAB4R in the fungistatic effect, but were not consistent in mitigating seed AF. Except for RAB24, they were also equal or even slightly superior to RAB4R in suppressing NOR synthesis (Figure 2), indicating that these RABs may have failed to stably colonize maize silks/ears (low “maize-phyllosphere competent”). For example, RAB6 is the common isolate chosen for field studies based on laboratory evaluation both in earlier work on rice [12] and the current studies in maize (Figure 3). Although the in vitro efficacy of RAB6 on rice sheath blight translated well in the field [12], it failed to replicate in maize.

### 3.2. RAB4R Provided Effective AF Biocontrol Both in Preharvest and Postharvest Infection Studies

RAB4R was not only effective in suppressing preharvest seed AF contamination (Figure 3) but was able also to restrict postharvest *A. flavus* infection and AF contamination of seeds (Figure 8). In addition to the four *A. flavus* strains used in fungistatic studies, anti-mycotoxigenic effects of RAB4R were successfully tested in highly-toxigenic strains from two predominant VCGs in Louisiana corn fields [20]. This demonstrated a broad and robust efficacy of RAB4R as an AF-BCA. Furthermore, AF reduction by RAB4R was often close to the lowest HPLC detection limits, suggesting that the isolate may be superior to atoxigenic *A. flavus* strains in keeping AF contamination within the regulated levels. Robust biocontrol of fungal diseases (e.g., rice blast) by other *B. methylotrophicus* isolates has been reported earlier, some being more effective than even commercial chemical fungicides [25].

### 3.3. RAB4R Inhibition of A. flavus Growth Involved Diffusible Anti-Fungal Compounds

RAB4R inhibited *A. flavus* growth by using secreted antifungal compounds that strongly compromised hyphal integrity and tip growth (Figure 4 and Figure 5). The manifestation of growth abnormalities depended on the proximity of *A. flavus* to RAB4R (≤0.5 cm), indicating the diffusion limits of secreted metabolites. The effects were conspicuous at the growing edge of the fungal colony, while the interior appeared unaffected with continued production of conidiophores. A partial recovery of growth in the inhibition zone was marked by an increased hyphal hydrophobicity and excessive branching (Figure 5C). Although these growth inhibitory effects are common to antifungals, including caspofungin or chemical fungicides ([23,24,25,32], Chalivendra, unpublished), the type and magnitude of changes in hyphal cells induced by RAB4R varied to some extent with the growth medium and *A. flavus* strain tested.

Methanol-extractable compounds (“LPs”) released into culture filtrates suppressed NOR production but they were not equally effective in inhibiting *A. flavus* growth (Figure 6). This may be because concentrations tested were suboptimal. Protein quantification by A_280_ measurement or the Bradford method was not precise. Most bioactive LPs are short and composed of as few as 3–5 amino acids that may not absorb UV or bind Coomassie Blue [14]. Many protein-rich fractions showed no activity and a few fractions with inhibitory activity had no measurable protein. Alternatively, fungistatic compounds made by RAB4R may not be methanol-extractable. *B. methylotrophicus* isolates are known to make both peptide and non-peptide antifungals, including volatiles [25,33,34,35].

### 3.4. RAB1 Showed Promising Anti-Aflatoxigenic Activity In Vitro and in Mature Maize Seeds

Although our initial intent was to use RAB1 as a negative control based on its lack of fungistatic activity, we observed that the *L. sphaericus* strain inhibits both NOR and AF synthesis in vitro (Figure 4) and in maize seeds (Figure 8). Hua et al. [19] also observed a similar lack of correlation between growth inhibition and reduction of NOR by yeasts. The anti-aflatoxigenic activity of RAB1 in vitro was effective from a greater distance than that of RAB4R (Figure 4), indicating a greater permeability or potency of the former. LPs isolated from RAB1 were also equally potent in suppressing NOR production (Figure 6). However, the isolate was less effective in the postharvest seed infection assay (Figure 8). This may be due to its poor fungistatic effect on *A. flavus* and the high susceptibility of the inbred Va35 for AF accumulation. RAB1 showed strong AF suppression when a commercial corn hybrid (Pioneer 2089YHR, DuPont, Wilmington, DE, USA) grown in Louisiana was tested in the assay (data not shown). *L. sphaericus* (synon. *B. sphaericus*) is better known for its insecticidal and nematicidal properties and a component of a commercial mosquitocide [36,37]. Nevertheless, the bacterium has also been successfully tested as a BCA against plant pathogens [38,39]. Another desirable feature of RAB1 is its lack of hemolytic activity (Figure 9), unlike RAB12 (Figure S4) and other *L. sphaericus* isolates [37], which may make the isolate safe to human handling and to non-target organisms [40].

### 3.5. In Vitro Studies Indicate the Potential of Other RABs as Independent AF-BCAs or as Helper BCAs in Combination with Intraspecific Biocontrol Strains

The fungistatic assay with the *nor* mutant (Figure 2) revealed that additional RABs deserve further analysis. Isolates, such as 17R, show both fungistatic and anti-mycotoxigenic activity superior to RAB4R and may prove to be highly effective alternative BCAs to atoxigenic *A. flavus* strains in field trials. A few others (e.g., RAB19) show strong suppression of NOR with little fungistasis and may provide robust AF-biocontrol as helper strains with atoxigenic *A. flavus* strains. Our future work will include testing the compatibility of these two systems together in the laboratory, as well as in field trials. Further, we plan to test the efficacy of RABs in controlling other common corn ear rot fungi, e.g., *Fusarium verticilloides*.

## 4. Conclusions

*Bacillus*-based BCAs are ideal for field application since the bacteria form spores and are amenable for formulation into stable products [14]. Our detailed studies on two of the RABs show their efficacy against multiple *A. flavus* strains that include highly-toxic local isolates as well as common laboratory strains. While RAB4R can mitigate AF contamination in the field as an independent BCA, RAB1 shows promise as a helper strain to the existing intraspecific AF-BCAs.

## 5. Materials and Methods

### 5.1. Strains and Media

All *A. flavus* strains used in the study were either obtained from the USDA Agricultural Research Service Culture Collection, Northern Regional Research Laboratory, Peoria, IL, USA (NRRL3357, 53, *A. flavus nor* mutant and AF36) or isolated from local corn fields (Tox4, VCG4, and VCG8; [20]). These strains have been used and described in more detail earlier [4,16,19,20]. Methods of isolation, culture, usage, and maintenance of RABs have been reported earlier [12]. Two solid media, potato-dextrose agar (PDA) and corn meal agar (CMA) were used in most of the studies and obtained from Hardy Diagnostics, Santa Maria, CA, USA. In a few studies, blood agar base (BAB; Difco Laboratories, Detroit, MI, USA) was also tested as described in **Results**. Blood agar plates (Remel Microbiology Products) were purchased from Thermo Scientific.

### 5.2. Measurement of Fungistatic Activity of Rabs

Four *A. flavus* strains, namely, 53, Tox4, *A. flavus nor*, and NRRL3357 were used in these studies. Approximately 5000 conidia (5 µL of 10^6^/mL) of each isolate were point-inoculated at the center of a PDA plate and RABs were inoculated using 10 µL of 10× concentrated overnight cultures [12] at the four corners of the plate 2.5 cm away from the center. Three plates were used per *Bacillus* strain and each *A. flavus* isolate was tested at least twice. The growth inhibitory zone (difference between the radii of *A. flavus* colony outside and below RAB colony) was measured and expressed as a percentage of the radius outside the RAB. If a RAB induced a large and merged inhibitory zone, the radius of *A. flavus* of the same strain grown in a control plate (where RABs were replaced by Luria-Bertani (LB) medium) was used for calculation. Tukey’s honest significant difference (HSD) test was used to analyze the data.

### 5.3. Field Evaluation of RAB Isolates for Reduction of AF Contamination in Maize

Based on growth inhibition data (Figure 1), the top five isolates that showed ≥60% growth inhibition were selected for field testing their AF biocontrol activities in maize (a seed corn hybrid, 25BHR26; Terral Seed, Rayville, LA, USA) during 2014 and 2015 in the LSU Experimental Station located in Baton Rouge, LA, USA. Tox4, a high AF-producing isolate from Louisiana maize fields, was used as the infection agent. RABs grown in LB for 3 d at 30 °C were pelleted and resuspended in sterile H_2_O at a density of 10^7^ cells/mL. At mid-silk stage, cell suspensions were sprayed on silks (1 mL/ear) in the middle two rows of four-row plots. Sterile water was sprayed in the negative control. Each treatment had four replicates. After 3 d, all treated plants were inoculated with silk sprays of Tox4 conidial suspensions (10^8^/mL). More than 20 ears were harvested from each plot at maturity, dried, and AF was estimated as described before [41]. Briefly, AF was extracted from 50 g of seed meal in 100 mL of 80% aqueous methanol. 1 mL aliquot of the filtered extract was passed through an activated basic alumina column to adsorb interfering pigments and used for AF determination by high-performance liquid chromatography (HPLC). The HPLC system and chromatography conditions were as detailed before [4].

### 5.4. Quantification of NOR

NOR was extracted from 13 mm agar plugs from the center and edges of the colony in alkaline methanol (90% methanol and 10% 1 N NaOH) and estimated spectrophotometrically by measuring the absorbance at 560 nm by as described in [19]. Three replicate plates were used per assay.

### 5.5. Microscopy

A stereomicroscope (Olympus BZ00, Tokyo, Japan) equipped with a digital camera (Dinoeye, Dunwell Tech, Inc., Torrance, CA, USA) and imaging software (Dinocapture, Dino-Lite, Dunwell Tech, Inc., Torrance, CA, USA) was used to image colony and hyphal morphology. Transmitted light was used to illuminate the samples.

### 5.6. Isolation and High Performance Liquid Chromatography (HPLC) Fractionation of Lipopeptides (Lps)

Cells from 1 L cultures of RAB1 and RAB4R grown in LB for 30 h at 30 °C were pelleted at 6000× *g* for 15 min and supernatants were collected. The pH of the cell-free medium was adjusted to 3.0 by a slow addition of 6 N HCl and stirring on ice. LPs were allowed to precipitate overnight by incubation at 4 °C and pelleted by centrifugation at 10,000× *g* for 30 min. The pellets were dissolved initially in 20–40 mL of methanol and 10-fold concentrated by flash evaporation at room temperature. The crude LP preps were separated in a Dionex ICS-3000 HPLC system (Thermo Fisher Scientific, Waltham, MA, USA), using a Protein and Peptide C18 column and a gradient 0.1% TFA in water and 0.1% TFA in acetonitrile at a flow rate of 1 mL/min. Peptides eluting at all major peaks of absorbance were collected, concentrated by freeze-drying and dissolving in 0.5 mL of methanol and stored at −20 °C until further use.

### 5.7. Bioassay of Lps for Biocontrol Activity

Total LPs and HPLC fractions were sterilized by passing through 0.2 µm microfilter. Fifty microliter aliquots of LPs or filter-sterile methanol (control) were spread at the center of PDA plates and spot-inoculated with *nor* mutant conidia. Plates were incubated at 30 °C for 3 d and photographed. NOR was estimated by extraction in alkaline methanol as described above. Average absorbance at 560 nm from three replicate plates is presented.

### 5.8. Analysis of Temperature and pH Stability of Total LP Extracts

The 0.5 mL aliquots of total LP extracts were incubated at 20, 30, 40, 50, or 60 °C for 30 min or autoclaved in a cycle of 50 min that exposes LPs to 121 °C, 15 Psi for 20 min to test the temperature stability of the biocontrol activities. To test the pH stability of LPs, the initial pH 3 was raised to pH 5, 7, 9, and 11 by adding 1 N NaOH to 0.5 mL aliquots. pH was monitored using narrow range pH strips. Final volume of pH adjusted samples was equalized to 600 µL. The biocontrol activity of all samples was analyzed using the *nor* mutant as described above. The temperature stability was expressed as a percentage of the activity at 20 °C and the pH stability as a percentage of activity at pH 3. The assays were repeated twice with three replicates at each pH and temperature.

## Supplementary Materials

The following supplementary figures are available online at http://www.mdpi.com/2072-6651/10/4/159/s1, Figure S1: RAB4R pretreated ears consistently showed low AF levels in *A. flavus* infected maize, Figure S2: HPLC elution profiles of lipopeptides isolated from RAB1 and RAB4R, Figure S3: RAB12, the other *L. sphaericus* isolate, shows hemolytic activity unlike RAB1.
