# Supplementary material for: Rice Phyllosphere Bacillus Species and Their Secreted Metabolites Suppress Aspergillus flavus Growth and Aflatoxin Production In Vitro and in Maize Seeds
Source: Toxins (Basel). 2018 Apr 16;10(4):159. doi: 10.3390/toxins10040159 (PMC5923325; doi:10.3390/toxins10040159)
Supplement: Supplementary file 1 [file toxins-10-00159-s001.pdf]

Supplementary Materials: Rice Phyllosphere Bacillus Species and Their Secreted Metabolites Suppress *Aspergillus flavus* Growth and Aflatoxin Production *in vitro* and in Maize Seeds

Subbaiah Chalivendra, Catherine DeRobertis, Jorge Reyes Pineda, Jong Hyun Ham and Kenneth Damann

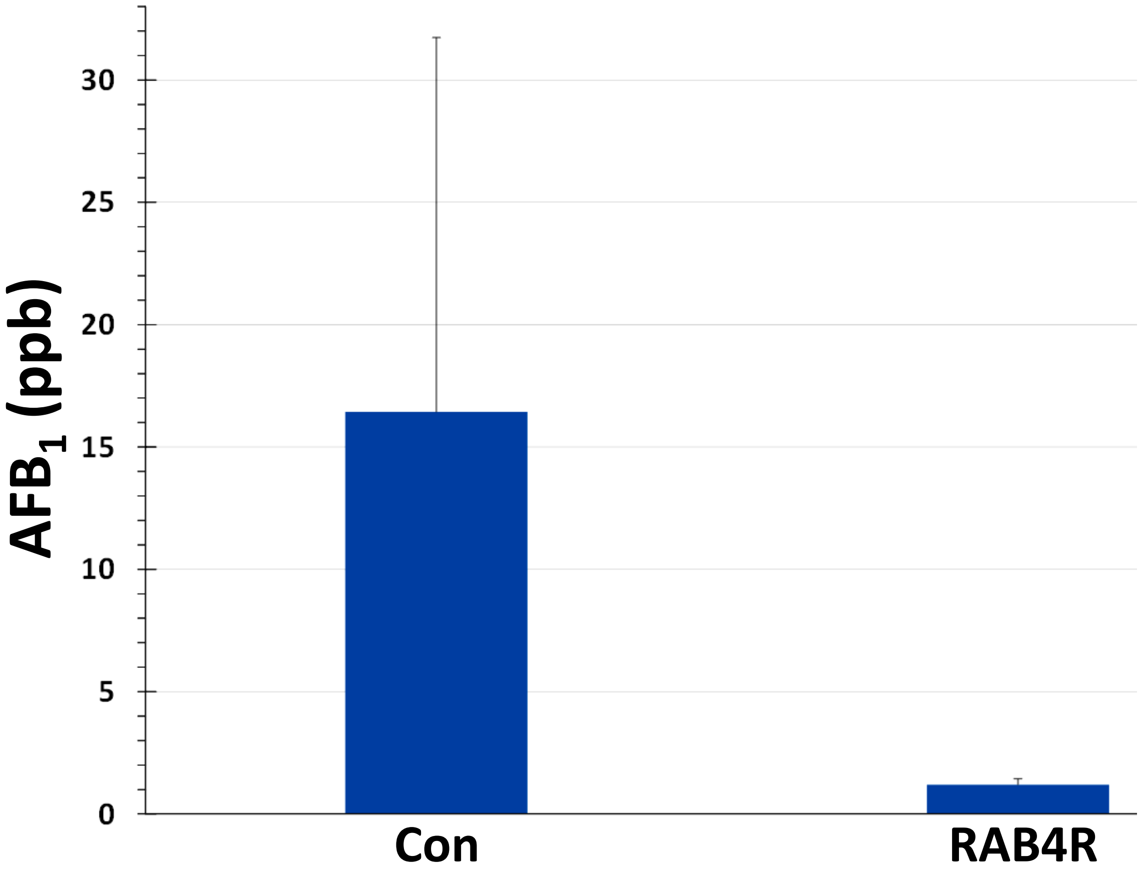

Figure S1. RAB4R pretreated ears consistently showed low AF levels in *A. flavus*-infected maize. Silks were pre-inoculated with RAB4R. After 3 d, the same silks were sprayed with conidia from *A. flavus* strain 53 as described in Material and Methods. AF values are the means + SE of 4 replicates (≥20 ears/rep).

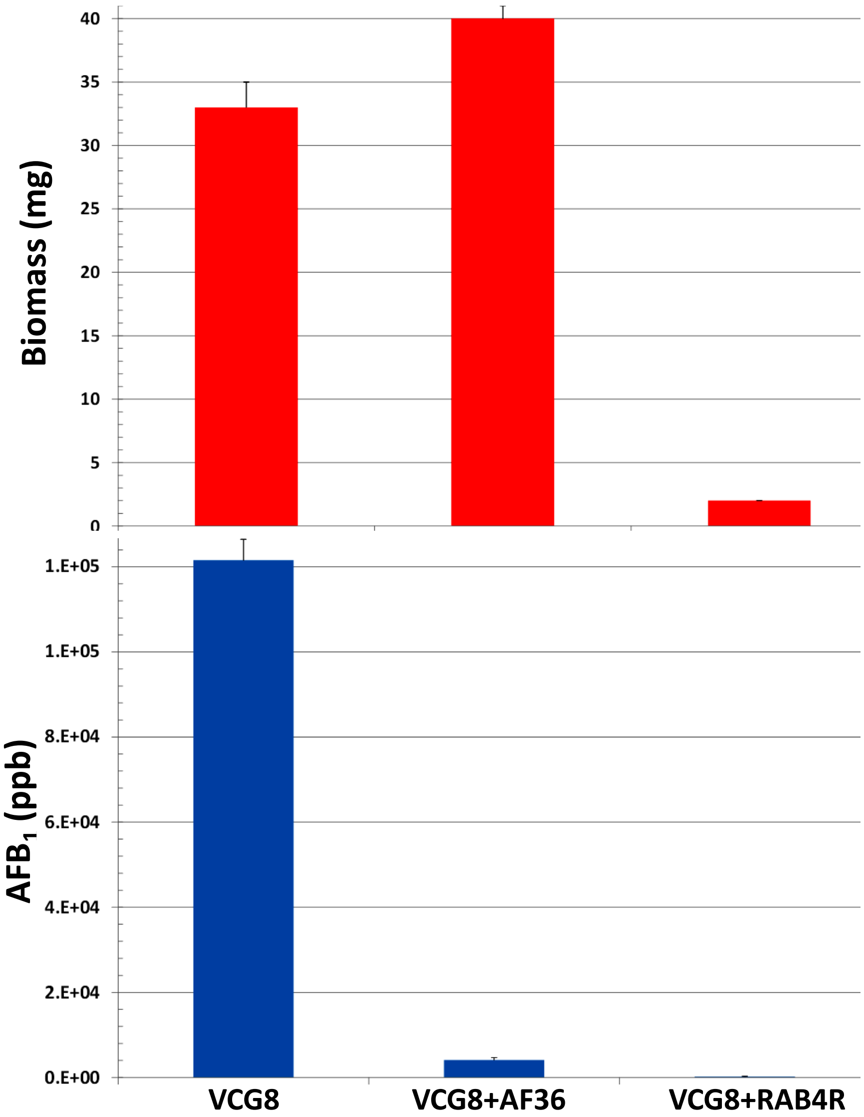

Figure S2. Comparison of RAB4R efficacy with that of AF36, an intraspecific biocontrol strain. Approximately 5000 conidia of the toxigenic strain VCG8, 5000 conidia each of VCG8 and AF36, or 5000 conidia of VCG8+5 μL of RAB4R overnight culture were added to 0.8 mL of YES medium in a 24-well plate and were grown at 30 °C for five days. AF was extracted in 80% methanol and determined by HPLC. The mycelium was collected, dried on a pre-weighed filter paper, and weighed in all treatments. Values are averages and +SE of three experiments with triplicate samples. AF values ranged between 10 and 23 ppb in VCG8+RAB4R, while they were 400 and 3600 ppb in VCG8+AF36 combination. Both mass and AF values were significantly different between treatments, with *p*-values ranging from 0.04 to <0.000001 as determined by Student's t test.

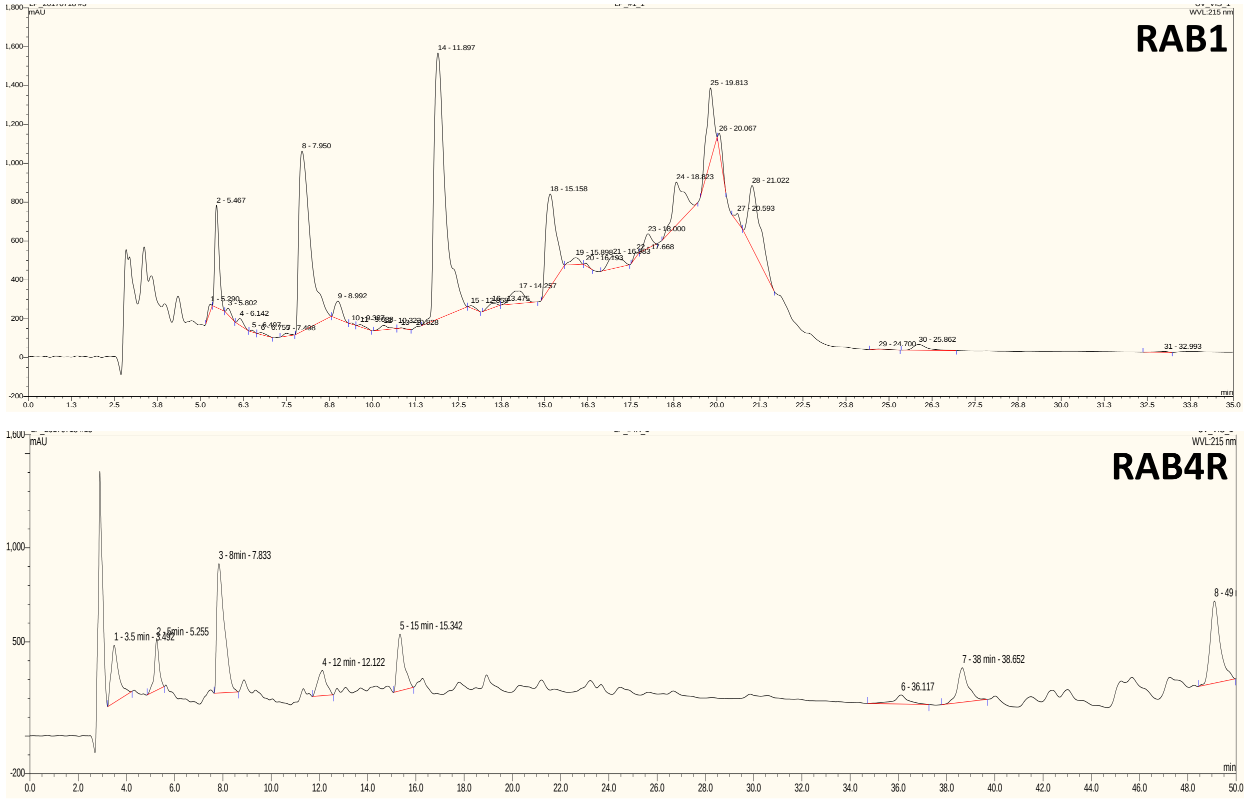

Figure S3. HPLC elution profiles of lipopeptides isolated from RAB1 and RAB4R. The chromatography details are given in the Materials and Methods.

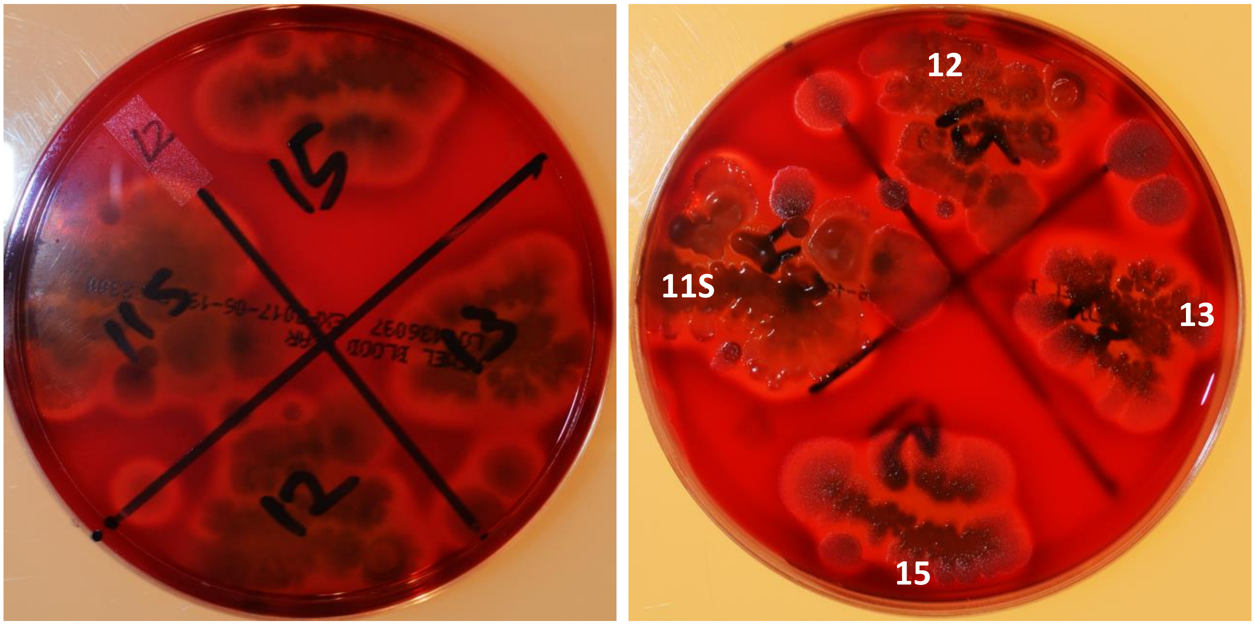

Top view **Bottom view**

Figure S4. The other *L. sphaericus* isolate RAB12 shows hemolytic activity. Hemolytic activity was tested using blood-agar plates as described in Figure 8.
